# Research on fine-tuning algorithms for Large Language Models integrating Uncertainty Modeling and External Memory Augmentation

**DOI:** 10.1371/journal.pone.0351493

**Published:** 2026-06-12

**Authors:** Yumeng Ma, Yue Xing, Di Wu, Yining Zhou, Yun Zi, Ming Wang, Yingnan Deng, Shuaidong Pan

**Affiliations:** 1 Arizona State University, Tempe, Arizona, United States of America; 2 University of Pennsylvania, Philadelphia, Pennsylvania, United States of America; 3 University of Southern California, Los Angeles, California, United States of America; 4 Texas A&M University, College Station, Texas, United States of America; 5 Georgia Institute of Technology, Atlanta, Georgia, United States of America; 6 Trine University, Phoenix, Arizona, United States of America; 7 Carnegie Mellon University, Pittsburgh, Pennsylvania, United States of America; University of Bergamo: Universita degli Studi di Bergamo, ITALY

## Abstract

This paper proposes a parameter-efficient fine-tuning framework that integrates uncertainty modeling with external memory augmentation, aiming to improve robustness, confidence calibration, and contextual completeness in downstream natural language processing tasks. From the methodological perspective, the uncertainty modeling module explicitly characterizes uncertainty in inputs and intermediate representations through feature-level estimation, cross-layer propagation, and confidence calibration, thereby enhancing training stability and reducing the influence of noisy signals. Meanwhile, the external memory augmentation module employs key-value retrieval and gated fusion mechanisms to provide reusable contextual support, alleviating information loss caused by limited contextual summarization and improving representation quality under heterogeneous evaluation settings. Extensive experiments and ablation studies were conducted on text classification and named entity recognition tasks across multiple public benchmark datasets, using GPT-2 Small, GPT-2 Medium, and LLaMA3-8B as backbone models. The results demonstrate that the proposed framework consistently outperforms several mainstream fine-tuning methods in terms of accuracy, F1 score, and robustness, while also showing stable behavior under learning-rate sensitivity and missing-information settings. Overall, this study provides a novel perspective for efficient and interpretable fine-tuning paradigms, achieving a favorable balance among performance improvement, parameter efficiency, and deployment feasibility, and offering a practical basis for future extensions to more complex downstream scenarios.

## 1. Introduction

With the rapid development of large language models (LLMs), parameter-efficient fine-tuning (PEFT) has become an important research direction for improving adaptability while reducing computational costs. Wang et al. [1] provided a methodological survey of PEFT for large models, summarizing approaches such as low-rank adaptation, prompt tuning, and modular extensions. Han et al. [2] systematically reviewed the advantages and limitations of different PEFT techniques in multi-task scenarios. These studies show that reducing parameter overhead while maintaining performance not only helps lower deployment costs but also lays the foundation for broader cross-domain applications of large models.

However, existing methods still face two prominent challenges. First, in uncertainty quantification, Vashurin et al. [3] introduced the LM-Polygraph benchmark framework, which revealed performance differences among uncertainty methods in large models. Catak et al. [4] proposed a convex hull-based quantification method, but it still suffers from instability in task transfer and long-tail distribution scenarios. Second, Niu et al. [5] pointed out that existing fine-tuning paradigms provide limited calibration at the functional level, making it difficult to address confidence imbalance under cross-task conditions. In addition, research on external memory augmentation has shown potential value. Wang et al. [6] proposed the LongMem framework, which integrates long-term memory into language models to alleviate context truncation and forgetting. Yet, how to efficiently combine memory mechanisms in parameter-efficient settings remains an open problem [7].

To address these limitations, this paper proposes a parameter-efficient fine-tuning framework that tightly couples uncertainty modeling with external memory augmentation under a unified optimization objective. The core contribution is not a simple parallel combination of two existing ideas, but a structured interaction mechanism in which uncertainty estimation is introduced as an explicit control signal for memory retrieval, fusion, and prediction calibration. In this framework, the uncertainty branch is responsible for producing token-level confidence features and sequence-level uncertainty summaries, while the memory branch uses these signals to regulate retrieval temperature, feature fusion strength, and decision conservativeness. Through this design, uncertainty modeling and memory augmentation are no longer independent auxiliary components, but mutually constrained modules that jointly participate in representation refinement and output calibration. This interaction mechanism improves the consistency between confidence estimation and contextual compensation, thereby providing a more coherent parameter-efficient fine-tuning framework for robust adaptation under noisy supervision, incomplete context, and cross-task distribution variation.

The main contributions of this work are as follows:

- A unified uncertainty-guided memory-augmented PEFT framework is developed, in which uncertainty estimation is directly embedded into the memory interaction process rather than being used only as a post-hoc confidence score. This design establishes an explicit coupling pathway between confidence modeling, memory retrieval, and prediction calibration.
- An uncertainty-controlled external memory mechanism is designed to regulate retrieval and fusion according to the current confidence state of the model. By allowing the uncertainty signal to adjust memory access and contextual integration, the framework improves the reliability of retrieved information under noisy and distribution-shifted conditions.
- A joint fine-tuning scheme is constructed to connect token-level uncertainty estimation, sequence-level uncertainty summarization, memory-based contextual compensation, and confidence-aware prediction generation within a single parameter-efficient pipeline. This integrated design yields consistent improvements on multiple benchmark tasks and demonstrates the practical value of coupling uncertainty control with internal memory enhancement.

## 2. Related work

### 2.1 Research progress in large language models

In recent years, the rapid development of large language models (LLMs) has become a key direction in natural language processing. Hoffmann et al. proposed strategies for compute-optimal training of large language models. They revealed the optimal balance between compute, model size, and data, which provides theoretical guidance for scaling models [8]. Based on this, Chowdhery et al. introduced the PaLM model. By using the Pathways framework, they enabled large-scale parallel training and demonstrated the strong capability of very large models in language modeling tasks [9]. These studies indicate that scaling and compute efficiency are the main driving forces for the progress of large language models.

With the development of open-source and foundation models, Touvron et al. released the LLaMA and LLaMA 2 series. They emphasized open and efficient design, providing reproducible and high-performance resources for the research community [10,11]. These models achieved strong performance on traditional language tasks and also promoted cross-domain applications of large language models. At the same time, Yin et al. presented a systematic survey of multimodal large language models. They summarized advances in text and image integration and highlighted the potential of LLMs in multimodal tasks [12].

On the other hand, many surveys have mapped the overall development of large language models. Minaee et al. provided a comprehensive overview that covers model architectures, applications, and challenges [13]. Hagos et al. reviewed the status and future trends of large language models from the perspective of generative artificial intelligence [14]. Zaza et al. focused on the latest advances in architectures and methodologies [15]. Han et al. summarized the evolution of architectures, optimization and compression, as well as application challenges [16]. These works show that large language models are moving from single-task adaptation toward multimodal and multi-domain integration. They also demonstrate significant potential in algorithmic optimization, resource utilization, and practical applications.

### 2.2. Advances in fine-tuning algorithms for large language models

The rapid development of large language models has driven the rise of parameter-efficient fine-tuning methods. Wang et al. analyzed the universality and limitations of prompt tuning. They revealed systematic differences in performance across tasks and model scales, providing theoretical guidance for later fine-tuning strategies [17]. At the same time, Zhang et al. proposed LLaMA-Adapter. It introduced zero-initialized attention to achieve a more efficient adaptation process [18]. Chavan et al. presented One-for-All LoRA, which extended low-rank adaptation into a general framework and showed significant advantages in multi-task scenarios [19]. These studies indicate that parameter efficiency and task adaptability are important directions in the development of fine-tuning algorithms.

In terms of methodological innovation, Runwal et al. proposed DEFT. By introducing sparsification into the PEFT framework, it reduced activation density and improved training efficiency [20]. Prottasha et al. presented Semantic Knowledge Tuning, which leveraged external semantic information to support parameter-efficient fine-tuning and improved downstream task performance [21]. Mahabadi et al. explored fine-tuning with multi-task shared hypernetworks, which enhanced parameter utilization efficiency across tasks [22]. These methods extend the boundary of parameter-efficient fine-tuning from single-task adaptation to cross-task and cross-domain applications.

Survey studies have also summarized the development of fine-tuning for large language models. Pratap et al. provided a systematic review of advanced fine-tuning techniques and summarized the trade-offs between performance and efficiency [23]. Hu et al. proposed the LLM-Adapters series, which offered modular expansion approaches for adapting large language models [24]. Chen et al. re-evaluated parameter-efficient tuning methods and identified their potential limitations [25]. In addition, Gao et al. introduced FashionGPT, a multi-LoRA adapter fusion method, which showed strong ability in instruction-tuning tasks [26]. Overall, these studies demonstrate that fine-tuning algorithms for large language models are evolving from simple efficiency exploration toward diversification and modularization. They focus not only on training and inference efficiency but also on generalization across tasks and real-world applications.

## 3. Method

This paper proposes a fine-tuning method for large language models that integrates Uncertainty Modeling (UM) and External Memory Augmentation (EMA). In the overall framework, the UM module is responsible for estimating confidence and quantifying uncertainty of model predictions. It provides reliability calibration during both training and inference, which enhances robustness and adaptability in complex tasks.

At the same time, the EMA module introduces an addressable external memory structure. This allows the model to dynamically retrieve and utilize external knowledge during inference, overcoming the limitations of relying only on internal parameter storage.

**Fig 1:**
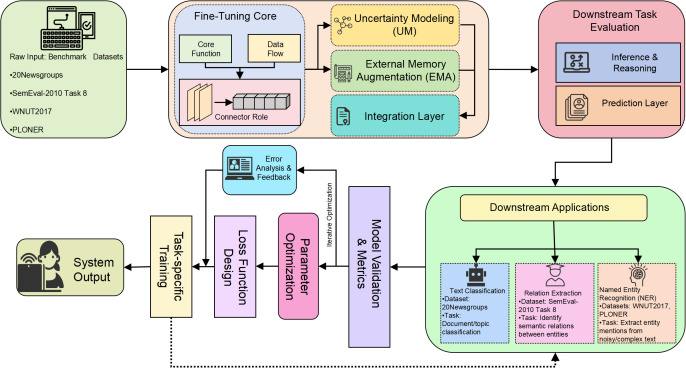
Overall framework of the proposed fine-tuning algorithm, which integrates Uncertainty Modeling and External Memory Augmentation to enhance reliability and generalization across downstream applications.

The two modules work together to improve efficiency and stability in fine-tuning. They also enhance the generalization ability of the model in cross-task and open environments. This provides a more scalable solution for the practical application of large language models. The overall framework diagram is shown in Fig 1.

### 3.1. Uncertainty modeling, UM

**Fig 2:**
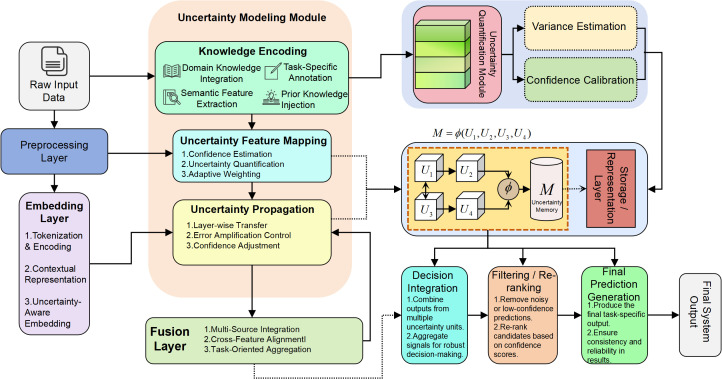
Architecture of the uncertainty modeling module. The module consists of guidance construction, uncertainty feature estimation, layer-wise uncertainty propagation, uncertainty quantification, and confidence calibration. It improves prediction reliability through internal feature interaction and structured uncertainty aggregation.

The uncertainty modeling module is designed to estimate prediction confidence at the token level and to provide uncertainty-aware calibration during fine-tuning. Its main purpose is to enhance robustness under noisy supervision and distribution shift while keeping the implementation path explicit. The overall framework is shown in Fig 2.

Given an input sequence


$$\begin{array}{c} X=\{x_{i}{\}}_{i=1}^{n},\;x_{i}\in {\mathbb{R}}^{d} \end{array}$$ (1)

the token embedding matrix is denoted by


$$\begin{array}{c} H=[\begin{array}{c} x_{1}^{⊤}\\ x_{2}^{⊤}\\ \vdots \\ x_{n}^{⊤} \end{array}]\in {\mathbb{R}}^{n\times d}. \end{array}$$ (2)

The guidance construction stage contains four clearly defined components, namely domain information, task information, semantic information, and prior information. To avoid ambiguity in implementation, these four components are all specified within the current training framework. The domain information is obtained by mean pooling over the current input sequence, which summarizes the overall contextual pattern of the sample. The task information is represented by a learnable task embedding indexed by the downstream task identity. The semantic information is obtained by max pooling over the token embedding matrix so as to preserve salient semantic responses. The prior information is represented by a shared trainable vector that serves as a global prior during optimization. Accordingly, the four components are defined as


$$\begin{array}{c} K_{d}=LayerNorm\;\left(\frac{1}{n}\sum_{i=1}^{n}x_{i}\right),\;K_{t}=e_{\tau },\;K_{s}=MaxPool(H),\;K_{p}=p_{0}. \end{array}$$ (3)

In this formulation, *K*_*d*_ and *K*_*s*_ are computed directly from the current input, *K*_*t*_ is determined by the known task identity, and *K*_*p*_ is optimized jointly with model training. This makes the source of each component explicit and keeps the construction process consistent across experiments. Each component is then projected into a common latent space and fused into a unified guidance representation:


$$\begin{array}{c} {\tilde{K}}_{r}={MLP}_{r}(K_{r}),\;\beta_{r}=\frac{\exp\;\left(w_{s}^{⊤}\tanh(W_{c}c+W_{r}{\tilde{K}}_{r})\right)}{\sum_{r^{\prime }}\exp\;\left(w_{s}^{⊤}\tanh(W_{c}c+W_{r^{\prime }}{\tilde{K}}_{r^{\prime }})\right)},\;E=\sum_{r\in \{d,t,s,p\}}\beta_{r}{\tilde{K}}_{r}, \end{array}$$ (4)

where


$$\begin{array}{c} c=LayerNorm\;\left(\frac{1}{n}\sum_{i=1}^{n}x_{i}\right). \end{array}$$ (5)

The role of this stage is to construct a compact guidance vector for uncertainty estimation rather than to introduce an additional prediction branch. The fused representation *E* summarizes contextual, task-related, semantic, and prior signals in a unified form, which is then used to modulate token-level confidence estimation.

In the uncertainty feature mapping stage, each token representation interacts with the fused guidance vector to produce an uncertainty-aware feature. The raw confidence score, quantized confidence, and final token-level uncertainty feature are computed by


$$\begin{array}{c} z_{i}=\tanh(W_{h}x_{i}+W_{e}E+b_{z}),\;u_{i}=\sigma (w_{u}^{⊤}z_{i}+b_{u}),\;q_{i}=\Delta \cdot round\;\left(\frac{u_{i}}{\Delta }\right), \end{array}$$ (6)

$$\begin{array}{c} \alpha_{i}=\frac{softplus(w_{\alpha }^{⊤}z_{i}+b_{\alpha })}{\frac{1}{n}\sum_{j=1}^{n}softplus(w_{\alpha }^{⊤}z_{j}+b_{\alpha })},\;U_{i}=\alpha_{i}q_{i}. \end{array}$$ (7)

Here, *u*_*i*_ denotes the initial confidence response of token *i*, *q*_*i*_ is a stabilized confidence value after quantization, and $\alpha_{i}$ is an adaptive importance coefficient used to normalize token contributions. Through this design, the module suppresses local fluctuations and retains more stable uncertainty signals for subsequent propagation. The initial uncertainty vector is written as


$$\begin{array}{c} U^{\left(0\right)}=[U_{1},U_{2},\ldots ,U_{n}{]}^{⊤}. \end{array}$$ (8)

In the uncertainty propagation stage, token-level uncertainty is further propagated through the hidden-state interaction structure of the backbone model:


$$\begin{array}{c} A^{\left(l\right)}=softmax\;\left(\frac{(H^{\left(l\right)}W_{Q}^{\left(l\right)})(H^{\left(l\right)}W_{K}^{\left(l\right)}{)}^{⊤}}{\sqrt{d_{a}}}\right),\;{\tilde{U}}^{\left(l\right)}=A^{\left(l\right)}U^{\left(l\right)}, \end{array}$$ (9)

$$\begin{array}{c} U^{\left(l+1\right)}=LayerNorm\;\left(U^{\left(l\right)}+\eta_{l}\tanh({\tilde{U}}^{\left(l\right)}W_{u}^{\left(l\right)}+b_{u}^{\left(l\right)})\right). \end{array}$$ (10)

This stage transfers uncertainty information across tokens and layers, allowing the model to capture inter-token dependency rather than treating each token independently. As a result, the uncertainty estimate reflects both local token confidence and global contextual interaction.

After layer-wise propagation, the module summarizes the uncertainty state and constructs a compact uncertainty memory:


$$\begin{array}{c} \bar{U}=\frac{1}{n}\sum_{i=1}^{n}U_{i}^{\left(L\right)},\;s_{U}=\frac{1}{n}\sum_{i=1}^{n}(U_{i}^{\left(L\right)}-\bar{U}{)}^{2}, \end{array}$$ (11)

$$\begin{array}{c} q_{m}=LayerNorm\;\left(W_{m}[c^{⊤},E^{⊤},\bar{U},s_{U}{]}^{⊤}+b_{m}\right),\;r_{j}=\frac{\exp(q_{m}^{⊤}k_{j})}{\sum_{\ell =1}^{J}\exp(q_{m}^{⊤}k_{\ell })}, \end{array}$$ (12)

$$\begin{array}{c} m=\sum_{j=1}^{J}r_{j}v_{j},\;M_{U}={MLP}_{M}([m^{⊤},\bar{U},s_{U}{]}^{⊤}). \end{array}$$ (13)

The uncertainty memory does not serve as an external knowledge source. Instead, it provides a compact representation of the propagated uncertainty state, making the confidence pattern reusable during calibration and decision integration. The statistics $\bar{U}$ and *s*_*U*_ respectively describe the average uncertainty level and its dispersion over the sequence.

The quantified uncertainty is decomposed into aleatoric and epistemic parts:


$$\begin{array}{c} \sigma_{q}^{2}=\frac{\Delta^{2}}{12},\;\sigma_{a}^{2}=\frac{1}{n}\sum_{i=1}^{n}U_{i}^{\left(L\right)}(1-U_{i}^{\left(L\right)})+\sigma_{q}^{2},\;\sigma_{e}^{2}=\frac{1}{n}\sum_{i=1}^{n}(U_{i}^{\left(L\right)}-\bar{U}{)}^{2}, \end{array}$$ (14)

$$\begin{array}{c} \sigma^{2}=\sigma_{a}^{2}+\sigma_{e}^{2}. \end{array}$$ (15)

The aleatoric term measures uncertainty induced by local confidence fluctuation and quantization, whereas the epistemic term reflects the dispersion accumulated after layer-wise propagation. Their combination provides a more complete uncertainty description for prediction calibration.

Finally, the module performs confidence calibration and generates the final prediction:


$$\begin{array}{c} T=softplus(w_{T}^{⊤}M_{U}+b_{T}),\;o_{i}=W_{o}h_{i}^{\left(L\right)}+b_{o}, \end{array}$$ (16)

$$\begin{array}{c} p_{i}=softmax\;\left(\frac{o_{i}-\lambda_{\sigma }\sigma^{2}1}{T}\right),\;\hat{Y}=\Omega \;\left(\{p_{i}{\}}_{i=1}^{n},E,M_{U},\sigma^{2}\right). \end{array}$$ (17)

The temperature parameter *T* is generated from the uncertainty memory so that the calibration strength adapts to the propagated uncertainty state. When the quantified uncertainty increases, the calibrated distribution becomes more conservative, which helps reduce overconfident predictions under difficult or noisy cases.

Overall, the uncertainty modeling module can be described as a sequential process consisting of guidance construction, uncertainty feature mapping, uncertainty propagation, uncertainty summarization, and confidence calibration. This formulation preserves the original modeling logic while making the definition of each information component, its construction path, and its functional role more explicit.

### 3.2. External memory augmentation, EMA

**Fig 3:**
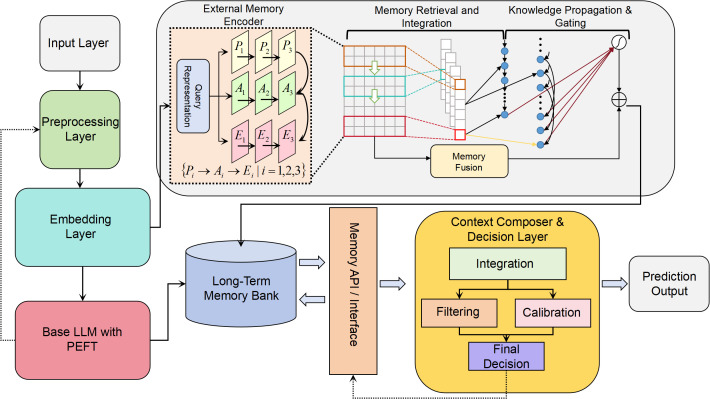
Overall architecture of the external memory augmentation module. The module uses a fixed-capacity key-value memory bank and performs query construction, uncertainty-aware retrieval, gated fusion, contextual decision adjustment, and continual memory update.

The external memory augmentation module is introduced to improve contextual completeness and prediction stability by storing reusable internal representations during fine-tuning. It receives the hidden states from the backbone model and the uncertainty signal from the UM branch, then performs memory retrieval, gated fusion, confidence-aware adjustment, and memory update. The overall framework is shown in Fig 3.

Let the backbone model produce the token-level hidden-state matrix


$$\begin{array}{c} H=[h_{1}^{⊤};\ldots ;h_{n}^{⊤}]\in {\mathbb{R}}^{n\times d},\;c=LayerNorm\;\left(\frac{1}{n}\sum_{i=1}^{n}h_{i}\right)\in {\mathbb{R}}^{d}, \end{array}$$ (18)

where *c* denotes the sequence-level contextual summary. The UM branch further provides the uncertainty memory embedding *M*_*U*_ and the quantified uncertainty scalar $\sigma^{2}$, which are used to regulate memory interaction.

The memory bank adopts a fixed-capacity key-value structure with *J* = 256 slots. For simplicity and implementation consistency, the key dimensionality and value dimensionality are both set equal to the hidden dimension, namely $d_{k}=d_{v}=d$. The memory bank is written as


$$\begin{array}{c} ℬ=\{(k_{j},v_{j}){\}}_{j=1}^{256},\;k_{j}\in {\mathbb{R}}^{d},\;v_{j}\in {\mathbb{R}}^{d}. \end{array}$$ (19)

At the beginning of training, all memory keys and values are initialized by Xavier uniform initialization. The memory bank is preserved across mini-batches and epochs within the same task, and is reinitialized when a new task starts. Therefore, the memory contents remain task-consistent and do not accumulate information across different task streams.

The current sample is encoded into a retrieval query by combining the contextual summary and the uncertainty embedding:


$$\begin{array}{c} q=LayerNorm\;\left(W_{q}[\begin{array}{c} c\\ M_{U} \end{array}]+b_{q}\right)\in {\mathbb{R}}^{d}. \end{array}$$ (20)

This design avoids introducing additional hierarchical query branches and keeps the retrieval input fully determined by the current hidden representation and the uncertainty state. The query is then matched against the fixed-capacity memory bank through scaled dot-product retrieval.


$$\begin{array}{c} s_{j}=\frac{q^{⊤}k_{j}}{\sqrt{d}},\;\alpha_{j}=\frac{\exp(s_{j}/\tau )}{\sum_{\ell =1}^{256}\exp(s_{\ell }/\tau )},\;m=\sum_{j=1}^{256}\alpha_{j}v_{j}, \end{array}$$ (21)

where the retrieval temperature is defined as


$$\begin{array}{c} \tau =softplus(w_{\tau }^{⊤}M_{U}+b_{\tau })+\lambda_{\tau }\sigma^{2}. \end{array}$$ (22)

The temperature term allows the retrieval sharpness to vary with the uncertainty level. When the uncertainty is higher, the matching distribution is softened to reduce the influence of unstable high-score slots. When the uncertainty is lower, the retrieval becomes more selective and emphasizes the most relevant memory items.

The retrieved memory is fused with the current representation through a gate:


$$\begin{array}{c} \gamma =\sigma \;\left(W_{\gamma }[\begin{array}{c} c\\ m\\ M_{U} \end{array}]+b_{\gamma }\right),\;z=\gamma ⊙m+(1-\gamma )⊙c. \end{array}$$ (23)

The gate vector γ controls the relative contribution of the retrieved memory and the current contextual summary. This prevents the memory branch from overwriting the backbone representation directly and ensures that the retrieved information only acts as a calibrated supplement. The fused contextual representation is then used for final prediction:


$$\begin{array}{c} C=LayerNorm(W_{c}z+b_{c}),\;\hat{Y}=\Omega (C). \end{array}$$ (24)

The memory update is performed once after each mini-batch forward pass. For each mini-batch, a new write key and a new write value are constructed from the current fused representation and uncertainty summary:


$$\begin{array}{c} k_{new}=LayerNorm(W_{k}z+b_{k}),\;v_{new}=LayerNorm(W_{v}[\begin{array}{c} C\\ \sigma^{2} \end{array}]+b_{v}). \end{array}$$ (25)

The insertion position is selected by a first-in-first-out queue rule. Specifically, the oldest slot in the bank is overwritten by (*k*_new_, *v*_new_) after each mini-batch update. This strategy keeps the storage cost fixed and avoids introducing an additional trainable replacement policy. The update frequency is therefore exactly once per mini-batch, and the effective memory always contains the most recent 256 contextual summaries observed in the current task. In the optimization process, the projection matrices, gating parameters, and prediction layers are all trained by standard backpropagation. The memory bank itself is treated as a dynamic buffer rather than a gradient-optimized parameter tensor. Consequently, gradients pass through the differentiable retrieval and fusion operations, while the slot replacement step is implemented as a non-differentiable buffer update after the forward computation. This design keeps the optimization process stable and makes the interaction between memory and backpropagation explicit. Accordingly, the EMA module is specified by the following concrete settings: a fixed memory size of 256 slots, key and value dimensions equal to the hidden size, Xavier uniform initialization, one update per mini-batch, persistence across epochs within the same task, reinitialization across tasks, and gradient-based optimization for all differentiable projections and fusion layers while treating memory slot replacement as a buffer operation. Under this formulation, the EMA branch provides a bounded and reproducible internal memory mechanism for contextual reuse and uncertainty-aware prediction enhancement.

## 4. Experiment

### 4.1. Datasets

#### 4.1.1. Text classification.

In the text classification task, two representative datasets were selected for experimental validation, namely 20Newsgroups and SemEval-2010 Task 8. The 20Newsgroups dataset, originally proposed by Lang [27], contains documents from 20 topic categories and is used in this study as a standard benchmark for multi-class text classification. For this dataset, each input sequence is encoded by the backbone model and then fed into a sequence-level classification head to predict a single category label, and the final performance is evaluated by classification accuracy. The SemEval-2010 Task 8 dataset, constructed by Hendrickx et al. [28], focuses on semantic relation classification between noun pairs and provides fine-grained annotated relation categories. In our implementation, the sentence containing the target entity pair is encoded as a whole and mapped to a unified sequence-level classifier for relation label prediction under the same evaluation protocol. These two datasets together provide complementary classification scenarios with different semantic granularity, thereby supporting a systematic evaluation of the proposed method in sequence-level prediction tasks.

#### 4.1.2. Named Entity Recognition (NER).

In the named entity recognition task, two widely used datasets, WNUT2017 and PLONER, were selected for validation. The WNUT2017 dataset, introduced by Derczynski et al. [29], contains noisy user-generated text and focuses on emerging and rare entities, while PLONER, adopted from the study of Fu et al. [30], provides a standard benchmark for token-level entity recognition under diverse annotation conditions. For both datasets, the model uses a token classification head built on top of the contextual token representations, and label prediction follows the original token-level annotation format of each dataset. During evaluation, predicted token labels are converted back to entity spans according to the standard BIO-style decoding rule, where a valid span *S* is formed by a beginning tag followed by all contiguous compatible inside tags of the same entity type, and the extracted spans are then compared with gold spans to compute the F1 score. Under this setup, the two datasets jointly provide a reliable basis for evaluating the proposed method on token-level structured prediction with consistent decoding and metric protocols.

### 4.2. Experimental setup

In the experimental setup, all comparisons were conducted under a unified software and hardware environment. GPT-2 Small (about 124M parameters, 12 layers), GPT-2 Medium (about 355M parameters, 24 layers), and LLaMA3-8B (about 8B parameters) were selected as backbone models. The evaluation focused on parameter-efficient fine-tuning paradigms. The implementation environment was Python 3.10 with PyTorch 2.1.0, running on two NVIDIA RTX 4090 GPUs with a total memory capacity of 48GB. CUDA and drivers were configured to match the platform for stable execution. To ensure comparability, training and evaluation protocols were kept consistent across four datasets: 20Newsgroups, SemEval-2010 Task 8, WNUT2017, and PLONER. The fine-tuning baselines included SplitLoRA, Lisa, MixLoRA, FLORA, ALoRA. For fairness, all baseline methods and the proposed method were trained under the same optimization settings within each backbone configuration, and only the method-specific parameter-efficient adaptation strategy was changed. The details are shown in Table 1.

**Table 1:** Descriptions of Parameter-Efficient Fine-Tuning Methods.

Method	Description
SplitLoRA	SplitLoRA proposes a split parameter-efficient fine-tuning framework. It allocates low-rank adaptation modules across different parts of the model to reduce both communication overhead and computation costs [31].
Lisa	Lisa is based on a layerwise importance sampling mechanism. It dynamically allocates updates across layers to improve storage efficiency in large model fine-tuning [32].
MixLoRA	MixLoRA introduces a mixture of low-rank adaptation strategy. It combines different low-rank representations in subspaces to achieve more efficient fine-tuning [33].
FLORA	FLORA focuses on improving cross-domain adaptation efficiency. It applies low-rank adaptation across heterogeneous domains to enable robust optimization under varying data distributions [34].
ALoRA	ALoRA proposes an adaptive low-rank allocation strategy. It assigns different ranks to adapters based on the needs of tasks and layers, improving flexibility and accuracy [35].

The sequence length was fixed at 512. Learning rate and batch size were adapted to the different backbones, including GPT-2 Small, GPT-2 Medium, and LLaMA3-8B, while remaining identical across all compared methods under the same backbone setting. The LoRA rank was selected from {4, 8, 16, 32}, and the main comparative experiments reported in this paper were conducted with rank *r* = 8 to balance efficiency and performance.

The optimization strategy employed AdamW with linear warm-up and gradient clipping to ensure stable convergence. Early stopping was applied based on development set F1 to prevent overfitting. The overall experimental setup is summarized in Table 2.

**Table 2:** Experimental setup and key hyperparameters.

Item	Setting
Backbone models	GPT-2 Small / GPT-2 Medium / LLaMA3-8B
Sequence length (tokens)	512
Batch size (per device)	8 (GPT-2 Small), 4 (GPT-2 Medium), 2 (LLaMA3-8B)
Learning rate (initial)	2 × 10^−4^
Optimizer	AdamW ($\beta_{1}=0.9,\;\beta_{2}=0.999$)
Weight decay / Gradient clipping	0.01 / 1.0
Learning rate schedule	Linear decay with 500-step warm-up
Training epochs (max)	5
Hardware and software	2 × RTX 4090, Python 3.10, PyTorch 2.1.0
Evaluation metrics	Accuracy, F1
Baseline	LoRA

### 4.3. Comparative experiment

#### 4.3.1. Text classification.

In text classification tasks, the proposed framework was systematically compared with mainstream parameter-efficient fine-tuning approaches, including SplitLoRA, Lisa, MixLoRA, FLORA, and ALoRA, under a unified experimental setting. To further verify the robustness and scalability of the proposed framework across different backbone capacities, experiments were conducted on GPT-2 Small, GPT-2 Medium, and LLaMA3-8B. The experimental results are reported in Table 3, where each result is presented as mean ± standard deviation over three runs with different random seeds.

**Table 3:** Performance comparison of parameter-efficient fine-tuning methods on text classification tasks. The reported results are mean accuracy (%) ± standard deviation over three runs with different random seeds.

Backbone	Method	20NEWS (Accuracy)			SEMEVAL (Accuracy)		
		200	500	1000	200	500	1000
GPT-2 Small	SplitLoRA	73.4±0.6	82.5±0.5	83.8±0.3	80.1±1.5	82.9±0.7	83.6±0.8
	Lisa	65.2±1.0	70.1±0.9	72.0±0.8	72.1±1.2	76.9±0.5	78.2±0.9
	MixLoRA	74.1±0.3	78.3±0.4	80.5±0.2	73.2±1.1	79.1±0.6	80.2±0.3
	FLORA	73.0±0.5	77.9±0.3	80.1±0.2	74.0±1.0	78.6±0.4	80.0±0.6
	ALoRA	77.1±0.6	80.2±0.6	82.3±0.5	80.7±0.6	83.0±0.5	83.8±0.6
	Ours	**78.6±0.5**	**82.8±0.4**	**84.7±0.3**	**81.9±0.4**	**84.2±0.5**	**85.1±0.4**
GPT-2 Medium	SplitLoRA	74.1±1.2	82.1±0.6	83.9±0.4	70.5±2.3	83.5±1.0	84.2±1.1
	Lisa	39.1±1.4	56.0±0.9	62.3±0.7	31.2±1.1	63.5±0.7	73.9±0.9
	MixLoRA	58.4±1.1	75.9±0.8	79.2±0.6	59.3±1.2	78.5±0.5	81.0±0.7
	FLORA	50.5±1.2	73.2±0.9	80.0±0.5	39.6±2.1	71.2±0.8	81.7±1.0
	ALoRA	73.9±1.1	82.5±0.7	84.3±0.6	72.4±2.2	83.0±0.8	83.4±0.5
	Ours	**79.6±0.5**	**83.8±0.4**	**85.0±0.3**	**80.9±0.4**	**84.7±0.5**	**85.8±0.4**
LLaMA3-8B	SplitLoRA	80.8±0.6	85.7±0.5	87.1±0.4	82.6±0.7	85.4±0.5	86.2±0.4
	Lisa	71.5±0.9	77.8±0.8	80.3±0.6	74.2±0.8	79.1±0.7	81.0±0.6
	MixLoRA	78.9±0.7	83.6±0.5	85.0±0.4	79.8±0.6	83.2±0.5	84.5±0.4
	FLORA	77.6±0.8	83.1±0.6	84.7±0.5	78.9±0.7	82.8±0.6	84.1±0.5
	ALoRA	81.9±0.5	86.2±0.4	87.6±0.3	83.4±0.5	86.1±0.4	86.9±0.3
	Ours	**83.7±0.4**	**87.5±0.3**	**88.9±0.2**	**85.2±0.4**	**87.6±0.3**	**88.4±0.2**

Table 3 shows that the proposed method consistently achieves the best performance across all backbone models, training scales, and datasets, demonstrating its strong effectiveness and stability in text classification tasks. Specifically, compared with existing parameter-efficient fine-tuning methods, Ours maintains clear advantages under both low-resource and relatively sufficient training settings, indicating that the proposed framework can better exploit limited supervision while preserving robust scalability as the number of training samples increases. In addition, the performance gains are observed not only on 20NEWS, which emphasizes topic-level discrimination, but also on SEMEVAL, which involves finer-grained semantic relation classification, suggesting that the method has good adaptability across different semantic granularities and task characteristics. It is also noteworthy that after introducing the stronger LLaMA3-8B backbone, Ours still remains consistently superior to all comparison methods, which further verifies that the proposed framework is not restricted to a specific model size and exhibits strong cross-backbone generalization ability. Meanwhile, the relatively small standard deviations obtained over three different random seeds indicate that the proposed method delivers stable optimization behavior and reliable performance reproducibility.

#### 4.3.2. Named Entity Recognition (NER).

Furthermore, the NER experimental results are presented, as shown in Table 4.

**Table 4:** Performance comparison of parameter-efficient fine-tuning methods on named entity recognition tasks. The reported results are mean F1 score (%) ± standard deviation over three runs with different random seeds.

Backbone	Method	WNUT2017 (F1)			PLONER (F1)		
		200	500	1000	200	500	1000
GPT-2 Small	SplitLoRA	49.0±0.8	51.5±0.7	53.0±0.6	62.5±0.9	65.0±0.7	66.8±0.6
	Lisa	50.8±0.7	52.6±0.6	54.7±0.5	64.0±0.8	66.2±0.6	68.1±0.5
	MixLoRA	51.2±0.7	53.0±0.6	55.2±0.5	64.5±0.8	67.0±0.6	68.7±0.5
	FLORA	50.5±0.8	52.3±0.6	54.5±0.5	63.8±0.8	66.5±0.6	67.9±0.5
	ALoRA	51.0±0.7	53.5±0.6	55.6±0.5	64.2±0.7	67.5±0.6	69.0±0.5
	Ours	**52.0±0.6**	**54.3±0.5**	**56.8±0.4**	**64.9±0.6**	**70.2±0.5**	**72.1±0.4**
GPT-2 Medium	SplitLoRA	50.2±0.9	52.0±0.7	53.8±0.6	63.0±0.9	65.8±0.7	67.5±0.6
	Lisa	51.5±0.8	53.2±0.6	55.0±0.5	64.3±0.8	67.1±0.6	68.9±0.5
	MixLoRA	51.9±0.8	53.7±0.6	55.8±0.5	64.8±0.8	67.8±0.6	69.5±0.5
	FLORA	51.0±0.8	53.0±0.6	55.2±0.5	64.0±0.8	67.2±0.6	69.0±0.5
	ALoRA	51.3±0.8	53.5±0.6	55.9±0.5	64.5±0.7	67.9±0.6	69.8±0.5
	Ours	**52.6±0.7**	**54.8±0.5**	**57.2±0.4**	**65.5±0.7**	**70.9±0.5**	**72.8±0.4**
LLaMA3-8B	SplitLoRA	54.8±0.7	57.1±0.5	58.9±0.6	68.2±0.8	71.0±0.6	72.6±0.5
	Lisa	53.7±0.9	56.0±0.7	57.8±0.4	67.1±0.7	69.9±0.8	71.5±0.6
	MixLoRA	55.3±0.6	57.8±0.8	59.6±0.5	68.7±0.9	71.6±0.5	73.2±0.7
	FLORA	54.5±0.8	57.0±0.6	58.7±0.7	67.9±0.6	71.1±0.7	72.8±0.5
	ALoRA	55.9±0.5	58.4±0.7	60.1±0.4	69.1±0.8	72.0±0.6	73.8±0.4
	Ours	**57.4±0.6**	**59.8±0.4**	**61.5±0.5**	**70.8±0.7**	**73.6±0.5**	**75.4±0.3**

Table 4 demonstrates that the proposed method consistently achieves the best F1 performance across all backbone models, data scales, and datasets, which verifies its effectiveness and robustness in named entity recognition tasks under both low-resource. Specifically, on WNUT2017, which contains sparse, noisy, and emerging entities from open domains, Ours maintains stable improvements over all competing parameter-efficient fine-tuning methods, indicating that the proposed framework has stronger adaptability to challenging entity distributions and better capability in handling noisy supervision. On PLONER, which further emphasizes long-tail characteristics, the superiority of Ours becomes even more evident, especially under the 500-shot and 1000-shot settings, showing that the method can more effectively capture discriminative entity representations while alleviating task interference during adaptation. In addition, with the introduction of the stronger LLaMA3-8B backbone, the proposed method still preserves the highest performance on both datasets, which further confirms that its advantages are not limited to a specific model scale and that it possesses strong cross-backbone generalization ability. Meanwhile, the relatively small yet non-uniform standard deviations over three different random seeds suggest that the proposed framework not only achieves reliable optimization stability, but also maintains good reproducibility under different random initialization conditions.

### 4.4. Ablation experiment

Building on the overall experiments, ablation studies were further conducted to gain deeper insights into the sources of performance improvement. By selectively removing or retaining key modules, the process provides a clear view of the marginal contributions of different mechanisms to model performance. At the same time, the ablation results not only help verify the independent value of each component in the proposed method but also offer solid evidence for understanding their synergy and guiding subsequent structural optimization.

#### 4.4.1. Text classification.

To further validate the effectiveness of different modules in the proposed method, ablation experiments were designed. By gradually incorporating the two core mechanisms, uncertainty modeling and external memory augmentation, it is possible to clearly observe the independent contribution of each module as well as the overall improvement when combined. This process not only verifies the rationality of each innovation but also highlights the importance of their synergy in optimizing model performance. The ablation results are shown in Table 5. For fair comparison, all reported results are presented as mean ± standard deviation over three runs with different random seeds.

**Table 5:** Ablation study results on text classification tasks. The reported results are mean accuracy (%) ± standard deviation over three runs with different random seeds.

Backbone	Variant	20NEWS (Accuracy)			SEMEVAL (Accuracy)		
		200	500	1000	200	500	1000
GPT-2 Small	Baseline	75.1±0.5	79.3±0.6	81.0±0.4	73.4±0.6	78.0±0.5	80.1±0.4
	+ UM	76.8±0.6	81.2±0.5	82.7±0.4	75.2±0.6	80.1±0.5	82.0±0.4
	+ EMA	77.3±0.5	81.7±0.4	83.2±0.6	75.8±0.5	80.7±0.4	82.6±0.6
	+ All (Ours)	**78.6±0.5**	**82.8±0.4**	**84.7±0.3**	**81.9±0.4**	**84.2±0.5**	**85.1±0.4**
GPT-2 Medium	Baseline	76.4±0.6	80.1±0.5	81.9±0.4	74.1±0.6	78.8±0.5	80.9±0.4
	+ UM	77.9±0.5	81.9±0.4	83.6±0.6	75.9±0.5	80.9±0.4	82.7±0.6
	+ EMA	78.5±0.4	82.3±0.6	84.1±0.5	76.4±0.4	81.3±0.6	83.2±0.5
	+ All (Ours)	**79.6±0.5**	**83.8±0.4**	**85.0±0.3**	**80.9±0.4**	**84.7±0.5**	**85.8±0.4**
LLaMA3-8B	Baseline	81.0±0.5	85.1±0.4	86.4±0.3	80.6±0.5	84.0±0.4	85.3±0.3
	+ UM	82.1±0.4	86.0±0.3	87.3±0.4	82.1±0.4	85.5±0.3	86.7±0.4
	+ EMA	82.6±0.6	86.5±0.5	87.8±0.3	82.8±0.5	86.0±0.4	87.2±0.3
	+ All (Ours)	**83.7±0.4**	**87.5±0.3**	**88.9±0.2**	**85.2±0.4**	**87.6±0.3**	**88.4±0.2**

The ablation results show that in text classification tasks, the gradual introduction of uncertainty modeling and external memory augmentation can significantly improve model performance. Uncertainty modeling dynamically weights input features in the representation space, allowing the model to remain robust when class boundaries are ambiguous, thereby improving overall accuracy. The further introduction of the external memory augmentation module enables the model to effectively aggregate contextual information across samples and tasks, addressing the limitations of single representations in long-text and semantic transfer scenarios. Finally, when both modules are applied together, the model achieves the best performance gains. This indicates that the two modules complement each other in global semantic modeling and fine-grained feature characterization, effectively enhancing generalization and discriminative ability in complex textual contexts.

#### 4.4.2. Named Entity Recognition (NER).

In the named entity recognition task, ablation experiments were conducted to further verify the independent contributions and synergistic effects of different modules in the proposed method. By gradually introducing uncertainty modeling and external memory augmentation, it is possible to systematically analyze the role of each module in improving entity recognition accuracy and robustness. This process not only reveals the effectiveness of individual modules but also demonstrates the performance advantages achieved when the two are combined. The ablation results are shown in Table 6. For fair comparison, all reported results are presented as mean ± standard deviation over three runs with different random seeds.

**Table 6:** Ablation study results on named entity recognition tasks. The reported results are mean F1 score (%) ± standard deviation over three runs with different random seeds.

Backbone	Variant	WNUT2017 (F1)			PLONER (F1)		
		200	500	1000	200	500	1000
GPT-2 Small	Baseline	50.0±0.6	50.5±0.4	53.4±0.8	62.0±0.5	65.6±0.2	65.8±0.9
	+ UM	50.9±0.5	52.8±0.6	53.9±0.6	63.2±0.7	66.4±0.2	67.8±0.6
	+ EMA	51.4±0.5	53.4±0.4	54.8±0.7	64.0±0.6	68.7±0.8	69.4±0.4
	+ All (Ours)	**52.0±0.6**	**54.3±0.5**	**56.8±0.4**	**64.9±0.6**	**70.2±0.5**	**72.1±0.4**
GPT-2 Medium	Baseline	50.8±0.5	52.6±0.4	54.5±0.7	63.8±0.4	66.7±0.6	68.1±0.6
	+ UM	51.2±0.8	53.5±0.6	55.7±0.5	64.0±0.8	67.7±0.6	69.2±0.5
	+ EMA	52.0±0.7	54.0±0.2	56.4±0.3	64.8±0.7	68.8±0.7	70.5±0.6
	+ All (Ours)	**52.6±0.7**	**54.8±0.5**	**57.2±0.4**	**65.5±0.7**	**70.9±0.5**	**72.8±0.4**
LLaMA3-8B	Baseline	55.1±0.8	57.2±0.5	59.0±0.6	68.0±0.6	71.2±0.4	72.9±0.7
	+ UM	55.8±0.4	58.1±0.7	60.0±0.5	69.0±0.8	72.1±0.6	73.7±0.4
	+ EMA	56.6±0.7	58.9±0.3	60.7±0.6	69.7±0.5	72.8±0.7	74.4±0.5
	+ All (Ours)	**57.4±0.6**	**59.8±0.4**	**61.5±0.5**	**70.8±0.7**	**73.6±0.5**	**75.4±0.3**

Table 6 shows that both uncertainty modeling and external memory enhancement contribute positively to named entity recognition performance across different backbones, sample scales, and datasets, while their combination achieves the best overall results. Specifically, compared with the baseline setting, introducing uncertainty modeling alone brings consistent improvements on both WNUT2017 and PLONER, indicating that modeling prediction uncertainty helps the framework better handle noisy entities and ambiguous boundary information under low-resource conditions. Meanwhile, the external memory enhancement module further improves F1 scores in most settings, especially on PLONER, suggesting that external memory provides useful complementary knowledge for entity recognition and long-tail entity discrimination. More importantly, when the two modules are integrated together, the proposed method consistently reaches the highest performance on all backbones, including GPT-2 Small, GPT-2 Medium, and LLaMA3-8B, which demonstrates a clear synergistic effect between adaptive uncertainty awareness and memory-based representation enhancement. In addition, the relatively small standard deviations over three different random seeds further indicate that the proposed framework maintains stable optimization behavior and reliable experimental reproducibility.

### 4.5. Experimental results of different learning rates

**Fig 4:**
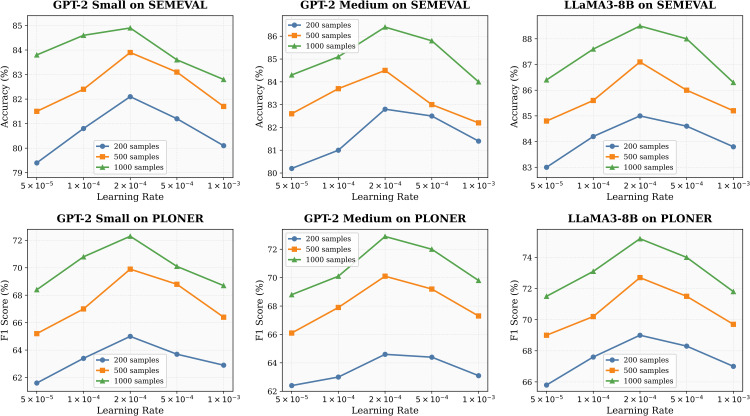
The figure illustrates the sensitivity of GPT-2 Small, GPT-2 Medium, and LLaMA3-8B to different learning rates on the SEMEVAL and PLONER tasks under 200, 500, and 1000 training-sample settings, showing that moderate learning rates generally yield the best performance across different backbone scales.

This paper also gives the experimental results of different learning rates, and the experimental results are shown in Fig 4.

The results in this figure show that all three backbone models exhibit clear sensitivity to the learning rate on both SEMEVAL and PLONER, and the best performance is generally achieved at moderate learning rates rather than at excessively small or large values. Specifically, across different sample sizes, the performance of GPT-2 Small, GPT-2 Medium, and LLaMA3-8B usually increases as the learning rate rises from 5 × 10^−5^ to around 2 × 10^−4^, but then declines when the learning rate is further enlarged to 5 × 10^−4^ and 1 × 10^−3^, indicating that overly aggressive updates may weaken optimization stability and generalization ability. In addition, larger training sample sizes consistently lead to better results under the same backbone and learning-rate setting, which confirms the positive effect of data scale on model adaptation. It is also noteworthy that LLaMA3-8B maintains the strongest overall performance on both tasks, while GPT-2 Medium generally outperforms GPT-2 Small, reflecting the advantage of stronger backbone capacity in low-resource fine-tuning scenarios. Overall, these observations suggest that selecting a proper intermediate learning rate is critical for achieving stable and competitive performance across different backbone scales and downstream tasks.

### 4.6. The impact of missing data on experimental results

**Fig 5:**
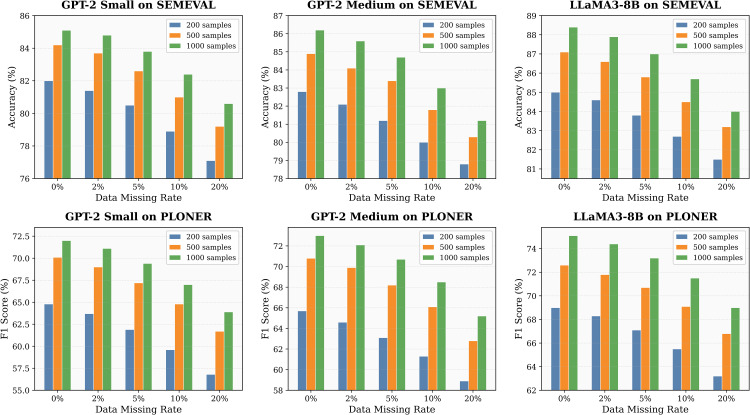
The figure illustrates the performance changes of GPT-2 Small, GPT-2 Medium, and LLaMA3-8B on the SEMEVAL and PLONER tasks under different missing data rates. The results show that the overall model performance decreases as the missing data rate increases, while larger-scale backbone models with more training samples generally have stronger robustness.

This paper also gives the impact of missing data on the experimental results, and the experimental results are shown in Fig 5.

The results in this figure show that model performance on both SEMEVAL and PLONER decreases progressively as the data missing rate increases, indicating that incomplete input information has a clear negative impact on downstream task effectiveness. Specifically, under all three backbone settings, the curves corresponding to 200, 500, and 1000 training samples exhibit a generally consistent downward trend, which suggests that the proposed framework remains sensitive to data integrity across different model scales and task types. At the same time, larger training sample sizes consistently achieve better results than smaller ones under the same missing-rate condition, demonstrating that richer supervision can partially alleviate the performance degradation caused by missing data. In addition, LLaMA3-8B maintains the strongest overall robustness on both tasks, while GPT-2 Medium generally outperforms GPT-2 Small, further confirming that stronger backbone capacity provides better resistance to information loss. Notably, the decline on PLONER is more pronounced than that on SEMEVAL, especially under high missing-rate settings, which suggests that named entity recognition may rely more heavily on complete contextual information than relation classification. Overall, these results verify that data missing rate is an important factor affecting model stability, and also highlight the advantage of larger backbones and richer training samples in improving robustness under incomplete-data conditions.

### 4.7. Uncertainty calibration analysis

**Fig 6:**
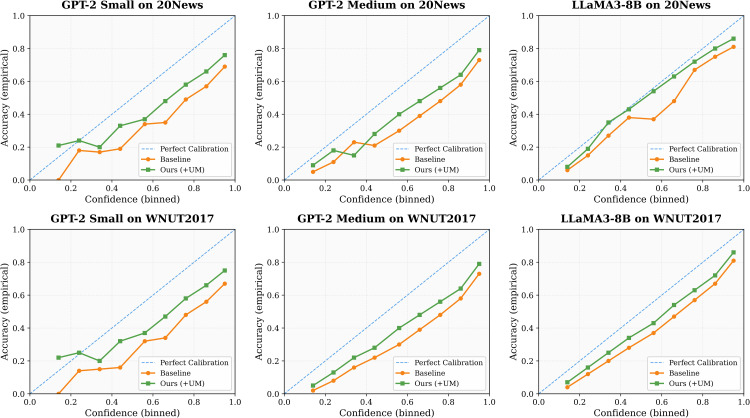
The figure shows the uncertainty calibration analysis results of GPT-2 Small, GPT-2 Medium and LLaMA3-8B on the 20News and WNUT2017 datasets. Ours (+UM) is closer to the ideal calibration curve than the baseline overall, indicating that the introduced uncertainty modeling mechanism can effectively improve the consistency between the model prediction confidence and the actual accuracy.

To further verify the effectiveness of the proposed method in predicting confidence calibration, this paper conducts uncertainty calibration analysis experiments. Specifically, two representative datasets, 20News and WNUT2017, are selected, and reliability diagrams are plotted on three backbone models: GPT-2 Small, GPT-2 Medium, and LLaMA3-8B, to compare the empirical accuracy performance of the baseline and the method after introducing uncertainty modeling in different confidence intervals. The experimental results are shown in Fig 6.

As illustrated in Fig 6, the proposed method consistently achieves better calibration behavior than the baseline across different backbone models and datasets, demonstrating the effectiveness of uncertainty modeling in improving the reliability of prediction confidence. Specifically, on both 20News and WNUT2017, the curve of Ours (+UM) is generally closer to the perfect calibration line, indicating that the predicted confidence is more aligned with the empirical accuracy after introducing uncertainty modeling. This advantage is particularly evident on the more challenging WNUT2017 dataset, where the baseline exhibits larger deviations from the ideal diagonal, while the proposed method shows a more stable and better-calibrated trend across different confidence intervals. In addition, as the backbone model becomes stronger from GPT-2 Small to GPT-2 Medium and further to LLaMA3-8B, the overall calibration quality is further improved, suggesting that larger models can provide more reliable confidence estimation when combined with the proposed framework. Overall, these results verify that the proposed method not only improves task performance, but also enhances the consistency between model confidence and actual correctness, which is crucial for trustworthy downstream deployment.

### 4.8. Calculation of overhead experimental results

**Fig 7:**
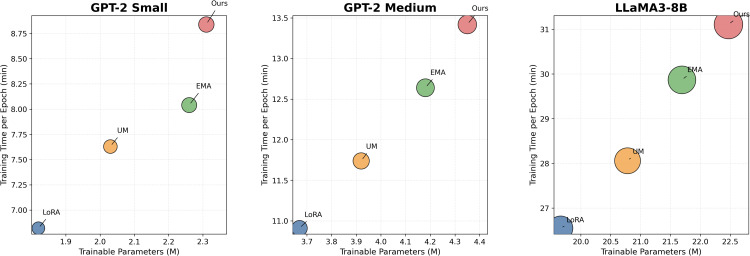
Comparison of computational overhead under different module configurations across three backbone models. The horizontal axis represents the number of trainable parameters, the vertical axis denotes the training time per epoch, and the bubble size reflects the corresponding GPU memory consumption.

To further evaluate the practical efficiency of the proposed framework, an additional analysis was conducted from the perspective of computational overhead under different module configurations and backbone scales. Specifically, the comparison focuses on trainable parameters, training time per epoch, and GPU memory consumption, so as to provide a clearer understanding of the cost variation introduced by UM, EMA, and their joint integration. The experimental results are shown in Fig 7.

Fig 7 shows that the computational overhead increases progressively as additional modules are introduced across all three backbone models, indicating that both UM and EMA inevitably bring extra training cost while remaining within a controllable range. Compared with the LoRA baseline, UM introduces a relatively moderate increase in trainable parameters and training time, whereas EMA leads to a more noticeable overhead growth, especially on the larger LLaMA3-8B backbone, which suggests that memory retrieval and update operations impose a stronger computational burden at larger model scales. Although the complete model achieves the highest overall overhead, its growth trend remains stable and does not exhibit an excessively sharp expansion, demonstrating that the joint integration of UM and EMA preserves a reasonable efficiency–performance trade-off while improving the functional completeness of the fine-tuning framework.

### 4.9. Quantitative Calibration Evaluation

To further quantitatively evaluate uncertainty calibration quality, we additionally report ECE, NLL, and Brier Score on all four datasets under the 1000-sample setting. As shown in Table 7, the proposed method consistently achieves lower calibration errors than the baseline across different backbone models and task settings, which provides complementary quantitative evidence to the reliability diagrams.

**Table 7:** Quantitative calibration evaluation under the 1000-sample setting. Lower values indicate better calibration. ECE denotes Expected Calibration Error, NLL denotes Negative Log-Likelihood, and Brier denotes Brier Score.

Backbone	Dataset	Variant	ECE↓	NLL↓	Brier↓
GPT-2 Small	20NEWS	Baseline	0.089±0.005	0.612±0.011	0.241±0.007
		Ours	**0.061±0.004**	**0.548±0.010**	**0.214±0.006**
	SEMEVAL	Baseline	0.097±0.006	0.648±0.013	0.256±0.008
		Ours	**0.066±0.005**	**0.571±0.012**	**0.226±0.007**
	WNUT2017	Baseline	0.143±0.007	0.981±0.017	0.332±0.010
		Ours	**0.109±0.006**	**0.887±0.015**	**0.296±0.009**
	PLONER	Baseline	0.118±0.006	0.802±0.015	0.287±0.009
		Ours	**0.089±0.005**	**0.724±0.013**	**0.254±0.008**
GPT-2 Medium	20NEWS	Baseline	0.081±0.005	0.586±0.010	0.229±0.006
		Ours	**0.055±0.004**	**0.522±0.009**	**0.203±0.005**
	SEMEVAL	Baseline	0.088±0.005	0.621±0.012	0.243±0.007
		Ours	**0.060±0.004**	**0.548±0.010**	**0.217±0.006**
	WNUT2017	Baseline	0.131±0.006	0.944±0.016	0.318±0.009
		Ours	**0.099±0.005**	**0.846±0.014**	**0.282±0.008**
	PLONER	Baseline	0.107±0.006	0.773±0.014	0.274±0.008
		Ours	**0.081±0.005**	**0.691±0.012**	**0.241±0.007**
LLaMA3-8B	20NEWS	Baseline	0.064±0.004	0.508±0.009	0.201±0.005
		Ours	**0.043±0.003**	**0.451±0.008**	**0.179±0.004**
	SEMEVAL	Baseline	0.071±0.004	0.537±0.010	0.214±0.006
		Ours	**0.048±0.003**	**0.476±0.009**	**0.191±0.005**
	WNUT2017	Baseline	0.112±0.005	0.865±0.014	0.287±0.008
		Ours	**0.086±0.004**	**0.781±0.013**	**0.255±0.007**
	PLONER	Baseline	0.091±0.005	0.701±0.012	0.248±0.007
		Ours	**0.068±0.004**	**0.633±0.011**	**0.221±0.006**

As shown in Table 7, the proposed method consistently achieves lower ECE, NLL, and Brier Score than the baseline across all backbone models and datasets, indicating that the uncertainty estimation and calibration mechanism improves the reliability of probabilistic predictions in a stable manner. From the perspective of task difficulty, the calibration errors on WNUT2017 are generally higher than those on 20NEWS and SEMEVAL, which suggests that noisy and sparse entity recognition scenarios pose greater challenges for confidence calibration, whereas the proposed method still maintains clear improvements under such settings. In addition, as the backbone scale increases from GPT-2 Small to GPT-2 Medium and further to LLaMA3-8B, both the baseline and the proposed method show progressively better calibration quality, while the full model consistently preserves an advantage over the corresponding baseline, demonstrating that the proposed framework can provide complementary calibration benefits across different model capacities and task types.

## 5. Conclusion

In this study, we proposed a unified framework that integrates Uncertainty Modeling with External Memory Augmentation under the scenario of parameter-efficient fine-tuning. UM explicitly estimates and propagates uncertainty at the representation level, suppressing noise and overfitting from both the gradient and confidence perspectives. Meanwhile, EMA leverages key-value memory retrieval to supplement contextual information and alleviate information loss caused by limited contextual summarization through gated fusion. Experiments conducted on text classification and named entity recognition tasks, based on GPT-2 Small, GPT-2 Medium, and LLaMA3-8B backbones with unified training and evaluation protocols, compared our method against multiple mainstream PEFT variants and included systematic ablation studies. The results demonstrate that UM enhances stability while EMA improves contextual representation quality, and their combination yields complementary benefits in most data settings. Additionally, learning-rate sensitivity experiments provide reusable guidance for hyperparameter tuning, while robustness analyses in missing-information scenarios further verify the framework’s applicability under noisy and incomplete inputs. To further complement the reliability diagrams, quantitative calibration evaluation was additionally conducted by introducing Expected Calibration Error (ECE), Negative Log-Likelihood (NLL), and Brier Score, which provides more direct evidence for the effectiveness of the proposed uncertainty calibration mechanism. Overall, the framework achieves a favorable balance among performance, stability, and interpretability with minimal parameter and engineering overhead, offering a reproducible baseline and methodological guidance for robust adaptation across diverse downstream task settings.

At the same time, the current study still has several limitations. First, the present evaluation is mainly conducted on discriminative tasks, including text classification and named entity recognition, which cannot fully reflect the potential of the proposed framework in generation-oriented application scenarios. More specifically, these tasks do not comprehensively test the behavior of the method under open-ended text generation, instruction following, or long-form content modeling, where memory-assisted contextual reuse and uncertainty-aware calibration may play a different role. Therefore, future work will further extend the evaluation to representative generative tasks such as summarization, instruction following, and other text generation settings, so as to examine whether the proposed framework can maintain its effectiveness under more typical GenAI-oriented applications. In addition, future research will continue to explore more lightweight and deployment-friendly designs, including trainable-parameter reduction, memory-efficient module construction, and latency–accuracy trade-off analysis, while also improving the engineering mechanism of the memory branch and validating the scalability of the framework on larger backbone models and more complex data settings.

## Supporting information

S1 File Supplementary tables. This file contains the fine-grained ablation results on text classification tasks, the fine-grained ablation results on named entity recognition tasks, and the full-parameter fine-tuning results.
(DOCX)

## References

[1] Wang L, Chen S, Jiang L, Pan S, Cai R, Yang S. Parameter-efficient fine-tuning in large models: A survey of methodologies. 2024. doi: arXiv:241019878

[2] Han Z, Gao C, Liu J, Zhang J, Zhang SQ. Parameter-efficient fine-tuning for large models: A comprehensive survey. arXiv preprint. 2024. doi: arXiv:240314608

[3] Vashurin R, Fadeeva E, Vazhentsev A, Rvanova L, Tsvigun A, Vasilev D. Benchmarking uncertainty quantification methods for large language models with LM-Polygraph. 2024. https://arxiv.org/abs/240615627

[4] Catak FO, Kuzlu M. Uncertainty quantification in large language models through convex hull analysis. Discov Artif Intell. 2024;4(1). doi: 10.1007/s44163-024-00200-w

[5] Niu R, Wu D, Yu R, Ma YA. Functional-level uncertainty quantification for calibrated fine-tuning on LLMs. In: 2024. https://arxiv.org/abs/241006431

[6] Cheng H, Dong L, Gao J, Liu X, Wang W, Wei F, et al. Augmenting Language Models with Long-Term Memory. In: Advances in Neural Information Processing Systems 36, 2023. 74530–43. 10.52202/075280-3259

[7] Marszałek P, Bałazy K, Tabor J, Kuśmierczyk T. Minimal ranks, maximum confidence: Parameter-efficient uncertainty quantification for LoRA. arXiv preprint. 2025. doi: arXiv:250212122

[8] Hoffmann J, Borgeaud S, Mensch A, Buchatskaya E, Cai T, Rutherford E. Training compute-optimal large language models. 2022. https://arxiv.org/abs/2203.15556

[9] Chowdhery A, Narang S, Devlin J, Bosma M, Mishra G, Roberts A. PaLM: Scaling language modeling with pathways. Journal of Machine Learning Research. 2023;24(240):1–113.

[10] Touvron H, Lavril T, Izacard X, Martinet X, Lachaux MA, Lacroix T, et al. LLaMA: Open and efficient foundation language models. arXiv preprint arXiv:230213971. 2023;.

[11] Touvron H, Martin L, Stone K, Albert P, Almahairi A, Babaei Y. LLaMA 2: Open foundation and fine-tuned chat models. arXiv preprint. 2023. doi: 10.48550/arXiv.230709288

[12] Yin S, Fu C, Zhao S, Li K, Sun X, Xu T, et al. A survey on multimodal large language models. Natl Sci Rev. 2024;11(12):nwae403. doi: 10.1093/nsr/nwae403 39679213 PMC11645129

[13] Minaee S, Mikolov T, Nikzad N, Chenaghlu M, Socher R, Amatriain X. Large language models: A survey. 2024. https://arxiv.org/abs/2402.06196

[14] Hagos DH, Battle R, Rawat DB. Recent Advances in Generative AI and Large Language Models: Current Status, Challenges, and Perspectives. IEEE Trans Artif Intell. 2024;5(12):5873–93. doi: 10.1109/tai.2024.3444742

[15] Zaza Z, Souissi O. Architectural and methodological advancements in large language models. Engineering Proceedings. 2025;97(1):8.

[16] Han S, Wang M, Zhang J, Li D, Duan J. A Review of Large Language Models: Fundamental Architectures, Key Technological Evolutions, Interdisciplinary Technologies Integration, Optimization and Compression Techniques, Applications, and Challenges. Electronics. 2024;13(24):5040. doi: 10.3390/electronics13245040

[17] Chauhan J, Hsieh C-J, Wang W, Wang Y. Universality and Limitations of Prompt Tuning. In: Advances in Neural Information Processing Systems 36, 2023. 75623–43. 10.52202/075280-3305

[18] Zhang R, Han J, Liu C, Gao P, Zhou A, Hu X. LLaMA-Adapter: Efficient fine-tuning of language models with zero-init attention. 2023. https://arxiv.org/abs/2303.16199

[19] Chavan A, Liu Z, Gupta D, Xing E, Shen Z. One-for-all: Generalized LoRA for parameter-efficient fine-tuning. 2023. https://arxiv.org/abs/2306.07967

[20] Runwal B, Pedapati T, Chen P-Y. From PEFT to DEFT: Parameter Efficient Finetuning for Reducing Activation Density in Transformers. AAAI. 2025;39(19):20218–27. doi: 10.1609/aaai.v39i19.34227

[21] Prottasha NJ, Mahmud A, Sobuj MSI, Bhat P, Kowsher M, Yousefi N, et al. Parameter-efficient fine-tuning of large language models using semantic knowledge tuning. Sci Rep. 2024;14(1):30667. doi: 10.1038/s41598-024-75599-4 39730415 PMC11681215

[22] Mahabadi RK, Ruder S, Dehghani M, Henderson J. Parameter-efficient multi-task fine-tuning for transformers via shared hypernetworks. arXiv preprint. 2021. doi: 10.48550/arXiv.2106.04489

[23] Pratap S, Aranha AR, Kumar D, Malhotra G, Iyer APN, S.S. S. The fine art of fine-tuning: A structured review of advanced LLM fine-tuning techniques. Natural Language Processing Journal. 2025;11:100144. doi: 10.1016/j.nlp.2025.100144

[24] Hu Z, Wang L, Lan Y, Xu W, Lim EP, Bing L, et al. LLM-adapters: An adapter family for parameter-efficient fine-tuning of large language models. 2023. doi: arXiv:230401933

[25] Chen G, Liu F, Meng Z, Liang S. Revisiting parameter-efficient tuning: Are we really there yet?. 2022. https://arxiv.org/abs/2202.07962

[26] Gao D, Ma Y, Liu S, Song M, Jin L, Jiang W, et al. FashionGPT: LLM instruction fine-tuning with multiple LoRA-adapter fusion. Knowledge-Based Systems. 2024;299:112043. doi: 10.1016/j.knosys.2024.112043

[27] Lang K. In: Machine Learning Proceedings 1995, 1995. 331–9.

[28] Hendrickx I, Kim SN, Kozareva Z, Nakov P, O Séaghdha D, Padó S. SemEval-2010 Task 8: Multi-way classification of semantic relations between pairs of nominals. In: 2019. https://doi.org/arXiv:191110422

[29] Derczynski L, Nichols E, van Erp M, Limsopatham N. Results of the WNUT2017 Shared Task on Novel and Emerging Entity Recognition. In: Proceedings of the 3rd Workshop on Noisy User-generated Text, 2017. 140–7. 10.18653/v1/w17-4418

[30] Fu J, Liu P, Zhang Q. Rethinking Generalization of Neural Models: A Named Entity Recognition Case Study. AAAI. 2020;34(05):7732–9. doi: 10.1609/aaai.v34i05.6276

[31] Lin Z, Hu X, Zhang Y, Chen Z, Fang Z, Chen X, et al. SplitLoRA: A split parameter-efficient fine-tuning framework for large language models. In: 2024. https://doi.org/arXiv:240700952

[32] Diao S, Han C, Liu X, Pan R, Pi R, Zhang J, et al. LISA: Layerwise Importance Sampling for Memory-Efficient Large Language Model Fine-Tuning. In: Advances in Neural Information Processing Systems 37, 2024. 57018–49. 10.52202/079017-1817

[33] Zhang J, Zhao Y, Chen D, Tian X, Zheng H, Zhu W. MiLoRA: Efficient mixture of low-rank adaptation for large language models fine-tuning. 2024. https://arxiv.org/abs/241018035

[34] He Y, Li A, Lyu L, Shen Z, Sun G, Wang H, et al. FLoRA: Federated Fine-Tuning Large Language Models with Heterogeneous Low-Rank Adaptations. In: Advances in Neural Information Processing Systems 37, 2024. 22513–33. 10.52202/079017-0708

[35] Liu Z, Lyn J, Zhu W, Tian X, Graham Y. ALoRA: Allocating low-rank adaptation for fine-tuning large language models. arXiv preprint. 2024. doi: 10.48550/arXiv.240316187

