# Supplementary material for: Research on fine-tuning algorithms for Large Language Models integrating Uncertainty Modeling and External Memory Augmentation
Source: PLoS One. 2026 Jun 12;21(6):e0351493. doi: 10.1371/journal.pone.0351493 (PMC13262865; doi:10.1371/journal.pone.0351493)
Supplement: S1 File — This file contains the fine-grained ablation results on text classification tasks, the fine-grained ablation results on named entity recognition tasks, and the full-parameter fine-tuning results. (DOCX) [file pone.0351493.s001.docx]

**Appendix**

**Table S1. Fine-grained ablation study on text classification tasks. Starting from the complete model, each variant removes one specific component from the uncertainty modeling (UM) or external memory augmentation (EMA) module. The reported results are mean accuracy (%) ± standard deviation over three runs with different random seeds.**

| **Backbone** | **Variant** | **20NEWS (Accuracy)** | | | **SEMEVAL (Accuracy)** | | |
| --- | --- | --- | --- | --- | --- | --- | --- |
|  |  | **200** | **500** | **1000** | **200** | **500** | **1000** |
| **GPT-2 Small** | w/o Guidance Construction | 77.5 ± 0.6 | 81.8 ± 0.5 | 83.5 ± 0.4 | 79.6 ± 0.5 | 82.3 ± 0.6 | 83.4 ± 0.5 |
|  | w/o Uncertainty Propagation | 77.1 ± 0.5 | 81.4 ± 0.6 | 83.1 ± 0.5 | 78.9 ± 0.6 | 81.7 ± 0.5 | 82.8 ± 0.4 |
|  | w/o Confidence Calibration | 77.8 ± 0.4 | 82.0 ± 0.5 | 83.8 ± 0.4 | 80.2 ± 0.5 | 82.8 ± 0.4 | 83.9 ± 0.5 |
|  | w/o Uncertainty-Aware Retrieval | 77.9 ± 0.5 | 82.1 ± 0.4 | 84.0 ± 0.5 | 80.4 ± 0.4 | 83.0 ± 0.5 | 84.0 ± 0.4 |
|  | w/o Gated Fusion | 77.3 ± 0.6 | 81.6 ± 0.5 | 83.4 ± 0.4 | 79.4 ± 0.5 | 82.0 ± 0.6 | 83.2 ± 0.5 |
|  | w/o Continual Memory Update | 78.0 ± 0.5 | 82.2 ± 0.4 | 84.1 ± 0.3 | 80.7 ± 0.4 | 83.3 ± 0.5 | 84.3 ± 0.4 |
|  | **Full (Ours)** | **78.6 ± 0.5** | **82.8 ± 0.4** | **84.7 ± 0.3** | **81.9 ± 0.4** | **84.2 ± 0.5** | **85.1 ± 0.4** |
| **GPT-2 Medium** | w/o Guidance Construction | 78.4 ± 0.5 | 82.8 ± 0.5 | 84.0 ± 0.4 | 79.2 ± 0.5 | 83.0 ± 0.6 | 84.1 ± 0.5 |
|  | w/o Uncertainty Propagation | 78.0 ± 0.6 | 82.4 ± 0.4 | 83.7 ± 0.5 | 78.6 ± 0.6 | 82.5 ± 0.5 | 83.8 ± 0.4 |
|  | w/o Confidence Calibration | 78.7 ± 0.4 | 83.0 ± 0.5 | 84.3 ± 0.4 | 79.8 ± 0.4 | 83.4 ± 0.4 | 84.5 ± 0.5 |
|  | w/o Uncertainty-Aware Retrieval | 78.8 ± 0.5 | 83.1 ± 0.4 | 84.4 ± 0.5 | 80.0 ± 0.5 | 83.6 ± 0.5 | 84.6 ± 0.4 |
|  | w/o Gated Fusion | 78.2 ± 0.5 | 82.6 ± 0.6 | 83.9 ± 0.4 | 79.0 ± 0.5 | 82.8 ± 0.6 | 84.0 ± 0.5 |
|  | w/o Continual Memory Update | 79.0 ± 0.4 | 83.2 ± 0.5 | 84.5 ± 0.4 | 80.2 ± 0.4 | 83.8 ± 0.5 | 84.8 ± 0.4 |
|  | **Full (Ours)** | **79.6 ± 0.5** | **83.8 ± 0.4** | **85.0 ± 0.3** | **80.9 ± 0.4** | **84.7 ± 0.5** | **85.8 ± 0.4** |
| **LLaMA3-8B** | w/o Guidance Construction | 82.6 ± 0.5 | 86.6 ± 0.4 | 87.9 ± 0.3 | 83.8 ± 0.5 | 86.5 ± 0.4 | 87.5 ± 0.3 |
|  | w/o Uncertainty Propagation | 82.2 ± 0.4 | 86.2 ± 0.5 | 87.5 ± 0.4 | 83.1 ± 0.4 | 85.9 ± 0.5 | 87.0 ± 0.4 |
|  | w/o Confidence Calibration | 82.9 ± 0.4 | 86.8 ± 0.3 | 88.1 ± 0.2 | 84.2 ± 0.4 | 86.8 ± 0.3 | 87.8 ± 0.3 |
|  | w/o Uncertainty-Aware Retrieval | 83.0 ± 0.5 | 86.9 ± 0.4 | 88.2 ± 0.3 | 84.5 ± 0.5 | 87.0 ± 0.4 | 87.9 ± 0.3 |
|  | w/o Gated Fusion | 82.4 ± 0.6 | 86.3 ± 0.4 | 87.6 ± 0.3 | 83.5 ± 0.5 | 86.1 ± 0.4 | 87.2 ± 0.3 |
|  | w/o Continual Memory Update | 83.1 ± 0.4 | 87.0 ± 0.3 | 88.3 ± 0.2 | 84.7 ± 0.4 | 87.1 ± 0.3 | 88.0 ± 0.2 |
|  | **Full (Ours)** | **83.7 ± 0.4** | **87.5 ± 0.3** | **88.9 ± 0.2** | **85.2 ± 0.4** | **87.6 ± 0.3** | **88.4 ± 0.2** |

**Table S2. Fine-grained ablation study on named entity recognition tasks. Starting from the complete model, each variant removes one specific component from the uncertainty modeling (UM) or external memory augmentation (EMA) module. The reported results are mean F1 score (%) ± standard deviation over three runs with different random seeds.**

| **Backbone** | **Variant** | **WNUT2017 (F1)** | | | **PLONER (F1)** | | |
| --- | --- | --- | --- | --- | --- | --- | --- |
|  |  | **200** | **500** | **1000** | **200** | **500** | **1000** |
| **GPT-2 Small** | w/o Guidance Construction | 51.1 ± 0.7 | 53.1 ± 0.5 | 55.2 ± 0.6 | 63.7 ± 0.6 | 68.8 ± 0.6 | 70.6 ± 0.5 |
|  | w/o Uncertainty Propagation | 50.8 ± 0.6 | 52.7 ± 0.6 | 54.7 ± 0.5 | 63.3 ± 0.7 | 68.2 ± 0.5 | 69.8 ± 0.6 |
|  | w/o Confidence Calibration | 51.3 ± 0.5 | 53.5 ± 0.4 | 55.6 ± 0.5 | 64.0 ± 0.5 | 69.1 ± 0.4 | 71.0 ± 0.5 |
|  | w/o Uncertainty-Aware Retrieval | 51.4 ± 0.6 | 53.6 ± 0.5 | 55.8 ± 0.4 | 64.2 ± 0.6 | 69.3 ± 0.5 | 71.2 ± 0.4 |
|  | w/o Gated Fusion | 50.9 ± 0.7 | 53.0 ± 0.6 | 55.0 ± 0.5 | 63.5 ± 0.7 | 68.5 ± 0.6 | 70.3 ± 0.5 |
|  | w/o Continual Memory Update | 51.5 ± 0.5 | 53.8 ± 0.4 | 56.0 ± 0.4 | 64.4 ± 0.5 | 69.6 ± 0.5 | 71.4 ± 0.4 |
|  | **Full (Ours)** | **52.0 ± 0.6** | **54.3 ± 0.5** | **56.8 ± 0.4** | **64.9 ± 0.6** | **70.2 ± 0.5** | **72.1 ± 0.4** |
| **GPT-2 Medium** | w/o Guidance Construction | 51.7 ± 0.7 | 53.7 ± 0.5 | 56.0 ± 0.5 | 64.3 ± 0.7 | 69.5 ± 0.6 | 71.3 ± 0.5 |
|  | w/o Uncertainty Propagation | 51.4 ± 0.6 | 53.3 ± 0.6 | 55.6 ± 0.5 | 64.0 ± 0.6 | 69.0 ± 0.5 | 70.8 ± 0.6 |
|  | w/o Confidence Calibration | 51.9 ± 0.5 | 53.9 ± 0.4 | 56.2 ± 0.4 | 64.5 ± 0.5 | 69.8 ± 0.4 | 71.6 ± 0.5 |
|  | w/o Uncertainty-Aware Retrieval | 52.0 ± 0.6 | 54.1 ± 0.5 | 56.4 ± 0.4 | 64.7 ± 0.6 | 70.0 ± 0.5 | 71.8 ± 0.4 |
|  | w/o Gated Fusion | 51.6 ± 0.7 | 53.5 ± 0.6 | 55.8 ± 0.5 | 64.1 ± 0.7 | 69.2 ± 0.6 | 71.0 ± 0.5 |
|  | w/o Continual Memory Update | 52.1 ± 0.5 | 54.2 ± 0.4 | 56.6 ± 0.4 | 64.9 ± 0.5 | 70.3 ± 0.5 | 72.0 ± 0.4 |
|  | **Full (Ours)** | **52.6 ± 0.7** | **54.8 ± 0.5** | **57.2 ± 0.4** | **65.5 ± 0.7** | **70.9 ± 0.5** | **72.8 ± 0.4** |
| **LLaMA3-8B** | w/o Guidance Construction | 56.4 ± 0.6 | 58.5 ± 0.5 | 60.6 ± 0.4 | 69.8 ± 0.6 | 72.7 ± 0.5 | 74.5 ± 0.4 |
|  | w/o Uncertainty Propagation | 56.1 ± 0.5 | 58.1 ± 0.6 | 60.2 ± 0.5 | 69.4 ± 0.7 | 72.3 ± 0.6 | 74.1 ± 0.5 |
|  | w/o Confidence Calibration | 56.6 ± 0.4 | 58.8 ± 0.4 | 60.8 ± 0.4 | 70.0 ± 0.5 | 72.9 ± 0.4 | 74.8 ± 0.4 |
|  | w/o Uncertainty-Aware Retrieval | 56.8 ± 0.5 | 59.0 ± 0.4 | 61.0 ± 0.3 | 70.2 ± 0.6 | 73.1 ± 0.5 | 75.0 ± 0.3 |
|  | w/o Gated Fusion | 56.3 ± 0.6 | 58.3 ± 0.5 | 60.4 ± 0.4 | 69.6 ± 0.6 | 72.5 ± 0.5 | 74.3 ± 0.4 |
|  | w/o Continual Memory Update | 56.9 ± 0.4 | 59.1 ± 0.3 | 61.1 ± 0.3 | 70.4 ± 0.5 | 73.2 ± 0.4 | 75.1 ± 0.3 |
|  | **Full (Ours)** | **57.4 ± 0.6** | **59.8 ± 0.4** | **61.5 ± 0.5** | **70.8 ± 0.7** | **73.6 ± 0.5** | **75.4 ± 0.3** |

**Table S3. Full parameter fine-tuning results**

| **Backbone** | **20NEWS / WNUT2017** | | | **SEMEVAL / PLONER** | | |
| --- | --- | --- | --- | --- | --- | --- |
|  | **200** | **500** | **1000** | **200** | **500** | **1000** |
| GPT-2 Small | 86.8 ± 0.4 | 90.2 ± 0.3 | 92.1 ± 0.2 | 89.6 ± 0.3 | 92.4 ± 0.2 | 93.6 ± 0.2 |
| GPT-2 Medium | 88.7 ± 0.3 | 91.8 ± 0.2 | 93.4 ± 0.2 | 91.1 ± 0.2 | 93.5 ± 0.2 | 94.6 ± 0.1 |
| LLaMA3-8B | 91.3 ± 0.2 | 93.8 ± 0.2 | 95.1 ± 0.1 | 93.2 ± 0.2 | 95.0 ± 0.1 | 96.1 ± 0.1 |
